# Fragmentation of decorin, biglycan, lumican and keratocan is elevated in degenerate human meniscus, knee and hip articular cartilages compared with age-matched macroscopically normal and control tissues

**DOI:** 10.1186/ar2453

**Published:** 2008-07-14

**Authors:** James Melrose, Emily S Fuller, Peter J Roughley, Margaret M Smith, Briedgeen Kerr, Clare E Hughes, Bruce Caterson, Christopher B Little

**Affiliations:** 1 Raymond Purves Research Laboratory, Institute of Bone & Joint Research, Kolling Institute of Medical Research, University of Sydney, Royal North Shore Hospital, Reserve Road, St. Leonards, NSW 2065, Australia; 2 Genetics Unit, 1529 Cedar, Rm 338, Shriners Hospital for Children, McGill University, Montreal, Quebec H3G 1A6, Canada; 3 School of Molecular and Medical Biosciences, PO Box 911, University of Cardiff, Cardiff CF1 3US, UK

## Abstract

**Introduction:**

The small leucine-rich proteoglycans (SLRPs) modulate tissue organization, cellular proliferation, matrix adhesion, growth factor and cytokine responses, and sterically protect the surface of collagen type I and II fibrils from proteolysis. Catabolism of SLRPs has important consequences for the integrity of articular cartilage and meniscus by interfering with their tissue homeostatic functions.

**Methods:**

SLRPs were dissociatively extracted from articular cartilage from total knee and hip replacements, menisci from total knee replacements, macroscopically normal and fibrillated knee articular cartilage from mature age-matched donors, and normal young articular cartilage. The tissue extracts were digested with chondroitinase ABC and keratanase-I before identification of SLRP core protein species by Western blotting using antibodies to the carboxyl-termini of the SLRPs.

**Results:**

Multiple core-protein species were detected for all of the SLRPs (except fibromodulin) in the degenerate osteoarthritic articular cartilage and menisci. Fibromodulin had markedly less fragments detected with the carboxyl-terminal antibody compared with other SLRPs. There were fewer SLRP catabolites in osteoarthritic hip than in knee articular cartilage. Fragmentation of all SLRPs in normal age-matched, nonfibrillated knee articular cartilage was less than in fibrillated articular cartilage from the same knee joint or total knee replacement articular cartilage specimens of similar age. There was little fragmentation of SLRPs in normal control knee articular cartilage. Only decorin exhibited a consistent increase in fragmentation in menisci in association with osteoarthritis. There were no fragments of decorin, biglycan, lumican, or keratocan that were unique to any tissue. A single fibromodulin fragment was detected in osteoarthritic articular cartilage but not meniscus. All SLRPs showed a modest age-related increase in fragmentation in knee articular and meniscal cartilage but not in other tissues.

**Conclusion:**

Enhanced fragmentation of SLRPs is evident in degenerate articular cartilage and meniscus. Specific decorin and fibromodulin core protein fragments in degenerate meniscus and/or human articular cartilage may be of value as biomarkers of disease. Once the enzymes responsible for their generation have been identified, further research may identify them as therapeutic targets.

## Introduction

Musculoskeletal disorders that affect the knee and hip represent a major cause of disability and morbidity in Western societies, exert a severe socioeconomic impact on afflicted individuals and are a heavy burden for health care resources [1-6]. Disruption of collagen fibres in articular cartilage and meniscus through the action of collagenolytic matrix metalloproteinases (MMPs) [7-9] and mechanical forces [10] represent a common end stage of musculoskeletal tissue disease. Numerous biosynthetic and catabolic events precede pathological collagen breakdown. Identifying changes in the extracellular matrix that not only precede collagen destruction but also predispose and lead directly to disease progression [11-13] may provide important targets for diagnosis and disease monitoring, and may facilitate early intervention strategies when the likelihood of therapeutic repair is enhanced.

The small leucine-rich proteoglycans (SLRPs) – including biglycan, decorin, fibromodulin, lumican and keratocan – play important linking, shape determining and matrix organizing roles [14-16]. These roles are essential for the correct functioning of musculoskeletal tissues such as the articular cartilages, which cover the ends of the long bones in the hip and knee, and fibrocartilages of the meniscus [17,18] and intervertebral disc. These tissues provide weight-bearing and tensile properties that are important for both joint articulation and the flexibility and mechanical stability of the appendicular skeleton. Menisci are semi-lunar fibrocartilages that lie on the superior tibial surface and improve its congruency with the curved femoral condylar surface. As such, the menisci are important stabilizing and weight-bearing structures in the knee joint [18]. With the onset of osteoarthritis (OA), the extracellular matrix of the menisci and articular cartilages undergo structural changes that are detrimental to their normal weight-bearing functional properties [18-22].

Direct evidence for the importance of the SLRPs in musculoskeletal tissues has been demonstrated using knockout mice. Although functional overlap between SLRP members is evident, a major phenotype of biglycan, decorin, fibromodulin and lumican single-knockout or double-knockout mice is age-dependent tendon laxity, ectopic calcification and arthritis [14,23-35]. We have recently shown that fragmentation of fibromodulin and biglycan compared with areas of intervertebral disc undergoing remodelling in an ovine annular lesion model of experimental disc degeneration [36].

The SLRPs have diverse functions in musculoskeletal tissues as modulators of tissue organization, cellular proliferation, matrix adhesion, and response to growth factors and cytokines (for review [37]). Importantly, the physical presence of the SLRPs on the surface of collagen type I and II fibrils can also sterically hinder the access of MMPs to the fibril and retard collagenolysis [11]. In light of their varied functions, catabolism of SLRPs is likely to have important consequences for the integrity of articular cartilage and meniscus by interfering with their homeostatic functions as well as physically exposing the collagen fibrils to enzymatic attack. To date, our knowledge about the proteinases responsible for SLRP proteolysis *in vivo *is very limited. Digests of purified or recombinant SLRPs have identified them as potential substrates for a variety of enzymes [38-42], but it is unclear whether the cleavages defined in vitro reflect physiologically relevant processes that actually occur in human tissue homeostasis or disease. Although changes with ageing in SLRP content and expression in bone and joint tissues have been well documented in humans [13,17,43-53], studies identifying SLRP proteolytic fragments in diseased human musculoskeletal tissues have thus far been restricted to arthritic knee articular cartilage [54-56]. It is unknown whether similar proteolysis of SLRPs occurs in degeneration/disease of all musculoskeletal tissues or in articular cartilages in all joints. The aim of this study was to evaluate and compare biglycan, decorin, lumican, fibromodulin and keratocan fragmentation in normal and degenerate human articular cartilages (hip versus knee) and meniscus.

## Materials and methods

### Tissues

This study was approved by the Human Research Ethics Committee of the Royal North Shore Hospital, St. Leonards, New South Wales, Australia. All tissues, normally discarded at surgery, were obtained with informed consent. Menisci (pooled medial and lateral tissue), knee (pooled femoral and tibial) and hip (femoral head) articular cartilage were obtained from patients undergoing total knee and hip replacements. Age-matched knee tissues (articular cartilage and meniscus) from six human cadaveric donors aged 60 to 75 years were obtained from The International Institute of Advancement in Medicine (Jessup, PA, USA; a division of the Musculoskeletal Foundation).

None of the donors had a history of OA or were on medication for degenerative joint disease. No severe articular cartilage erosion, osteophytosis or structural abnormalities were apparent on visual inspection of the joints at dissection, other than expected mild articular cartilage surface fibrillation in the region of the tibial plateau not covered by the meniscus. Articular cartilage was sampled separately from macroscopically 'normal' and mildly surface fibrillated cartilage regions from the 60- to 75-year-old cadaveric donors; these are referred to in this report as normal age-matched and fibrillated articular cartilage to distinguish these tissue samples from the more degenerate articular cartilage sampled from the total knee replacement femoral and tibial cartilages, which are referred to as OA articular cartilage. Similarly menisci from the 'normal' (non-OA) 60- to 75-year-old cadaveric donors were referred to as 'normal' menisci to distinguish these from menisci sampled from total knee replacement donors, which contained degenerate OA articular cartilage; these latter tissues are referred to as OA menisci because they contained degenerate fibrillated and/or torn regions and macroscopically damaged peripheral regions. Age-matched normal young knee articular cartilage from two 29-year-old specimens was obtained with ethical approval at the time of autopsy from the pathology departments at Montreal General Hospital, Montreal, Quebec, Canada.

### Antibodies

A number of affinity purified rabbit polyclonal antibodies to the carboxyl-terminal peptide sequences of decorin, biglycan, fibromodulin and lumican, and a monoclonal antibody to keratocan core protein were used in this study [57]; details of these are provided in Table 1.

**Table 1:** Peptide sequences identified by the SLRP antibodies used

SLRP (antibody type)	Peptide sequence identified	Antibody
Decorin (polyclonal)	(CGG)YVRSAIQLGNYK	PR-84
Biglycan (polyclonal)	(CGG)TDRLAIQFGNYKK	PR-85
Fibromodulin (polyclonal)	(CGG)LRLASLIEI	PR 184
Lumican (polyclonal)	(CGG)LRVANEVTLN	PR-353
Keratocan (monoclonal)	keratocan core protein (epitopes not identified)	KER-1

KER, keratocan; SLRP, small leucine rich proteoglycan.

### Extraction of tissues

Tissues were cut into small pieces using scalpels and extracted with 10 volumes of 4 M GuHCl 0.5 M sodium acetate (pH 5.8) containing 10 mmol/l EDTA, 20 mmol/l benzamidine and 50 mmol/l 6-aminohexanoic acid using end-over-end mixing for 48 hours at 4°C. The tissue residues were separated from the extracts by centrifugation and discarded. An aliquot of the 4 M GuHCl tissue extracts from each individual was pooled to generate a representative extract of the different tissues, and subjected to centrifugal diafiltration over a 100 kDa membrane and the diafiltrate (< 100 kDa) concentrated over a 5 kDa cut-off membrane. The 5 to 100 kDa fraction so obtained was dialyzed against three changes of milliQ (Millipore, N. Ryde, NSW, Australia) water and freeze dried. The remaining tissue extracts from individual donors/tissues were similarly dialyzed but were not fractionated by centrifugal diafiltration.

### Chondroitinase ABC and keratanase-I digestion of tissue extracts

Freeze dried tissue extracts were re-dissolved (2 mg dry weight/ml) overnight in 100 mmol/l Tris 0.03 M acetate buffer (pH 6.5) at 4°C with constant end-over-end mixing, and aliquots (0.5 ml) were digested with chondroitinase ABC (0.1 U) and keratanase-I (0.05 U) overnight at 37°C.

### Lithium dodecyl sulphate PAGE and detection of SLRP fragments by Western blotting

Aliquots of the chondroitinase ABC, keratanase-I digested samples (0.1 ml) were mixed with 4 × lithium dodecyl sulphate PAGE application buffer (35 μl) and 500 mmol/l dithiothreitol (15 μl). The samples were then heated at 70°C for 30 minutes, cooled, and 25 μl aliquots were electrophoresed under reducing conditions on 10% NuPAGE Bis-Tris gels at 200 V constant voltage for 50 minutes using NuPAGE MOPS (3- [N-morpholino]-propanesulfonic acid) sodium dodecyl sulphate running buffer. The gels were electroblotted to nitrocellulose membranes (0.22 μm) using NuPAGE transfer buffer supplemented with 10% methanol at 30 V constant voltage for 1 hours. SeeBlue-2 prestained protein molecular weight standards (InVitrogen Australia, Mount Waverley, Vic, Australia) were also electrophoresed for molecular weight calibration and to assess the blotting transfer efficiency.

The blots were initially blocked for 3 hours with 5% bovine serum albumin in 50 mmol/l Tris-HCl 0.15 M NaCl (pH 7.2; TBS) and then rabbit affinity purified anti-carboxyl-terminal antibodies (0.3–1 μg/ml) and anti-keratocan hybridoma conditioned media (KER-1; 1/100 dilution) were added overnight in 2% bovine serum albumin in TBS. After a brief rinse in TBS, goat anti-rabbit or anti-mouse IgG alkaline phosphatase conjugates (as appropriate) diluted in TBS (1/5,000 dilution) were then added, and after a further 1 hour the blots were washed in TBS (3 × 10 min). Then, NBT/BCIP (nitro-blue tetrazolium chloride/5-bromo-4-chloro-3'-indolyphosphate) substrates were added in alkaline phosphatase development buffer (0.1 M Tris-HCl [pH 9.5] containing 5 mmol/l MgCl2) for detection of immune complexes. Colour development was allowed to proceed for 20 minutes at room temperature and then the blots were rinsed in milliQ distilled water and dried. Western blots were repeated a minimum of three times, and the blots presented are representative of these. Blots were also conducted omitting primary antibody to check that no IgG species were present in the tissue extracts that crossreacted with the conjugated secondary detection antibodies; no false positive bands were detected (data not shown).

## Results

**Figure 1:**
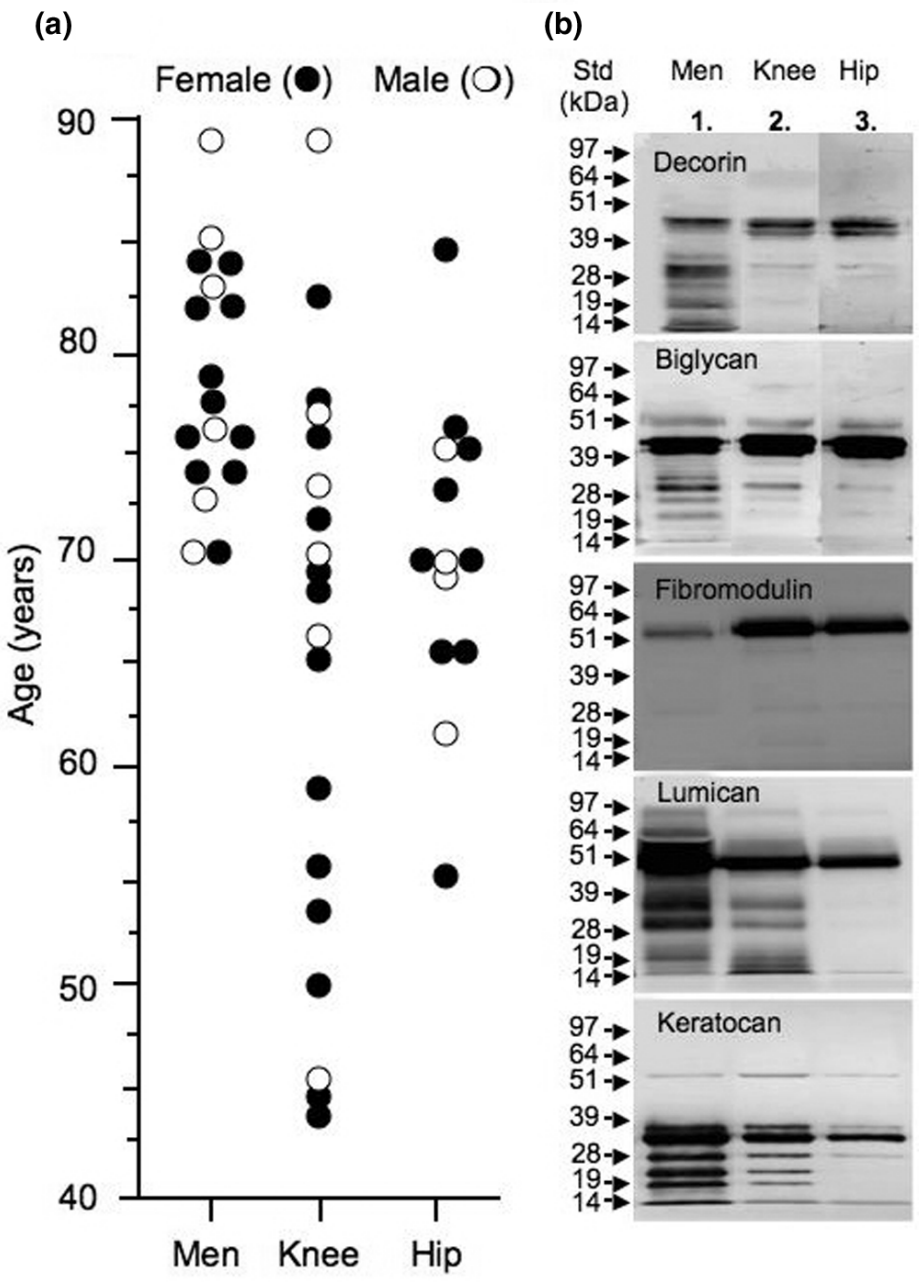
Assessment of SLRP fragmentation. Presented is an assessment of small leucine-rich proteoglycan (SLRP) fragmentation in human meniscus (Men), knee and hip articular cartilage extracts by Western blotting. **(a) **The age and sex distribution of the total knee and hip replacement tissue donors used in this study. **(b) **Pooled tissue extracts were examined by Western blotting. Pooled 4 M GuHCl tissue extracts were fractionated by centrifugal diafiltration and the 5 to 100 kDa fraction used. All samples were pre-digested with chondroitinase ABC and keratanase-I before electrophoresis. Sample loadings were normalized by loading extracts corresponding to equivalent wet weights of tissue in each lane for comparison.

Female patients predominated in all donor groups/tissues (60% to 70%) used in this study, which is consistent with the higher incidence of OA in the ageing female population (Figure 1a). The knee and hip articular cartilage donor groups ranged in age from 43 to 88 years (mean ± standard deviation [SD]: 68.6 ± 10.5 years) and from 55 to 85 years (mean ± SD: 69.8 ± 7.4 years), respectively, and the meniscal group ranged in age from 70 to 88 years (mean ± SD: 77.8 ± 5.4 years). The mean age of the meniscal donors used in this study was significantly older than all other sample groups (*P *< 0.006 for all analyses).

To try and compare all SLRPs in representative samples of different tissues, we pooled an aliquot of all 4 M GuHCl extracts from like tissues. In these pooled samples we depleted aggrecan species from samples destined for immunoblotting to avoid possible interference with sample concentration and electrophoretic separation of the SLRPs, by using centrifugal diafiltration. These pooled tissue extracts exhibited significant fragmentation of decorin, biglycan, lumican and keratocan in all tissues examined in the study (Figure 1b), but importantly the extent of fragmentation varied with SLRP, tissue type and joint (knee versus hip). Fibromodulin was not as extensively processed as the other SLRPs in the degenerate tissues initially examined in this study (Figure 1b). Meniscal extracts generally contained the most extensive range of SLRP fragments, and hip articular cartilage the least extensive fragmentation patterns. There was a marked difference between OA knee and hip articular cartilage in the fragmentation of lumican and keratocan but not biglycan, decorin or fibromodulin, despite similar levels of intact core proteins of the SLRPs in the two articular cartilages (Figure 1b).

**Figure 2:**
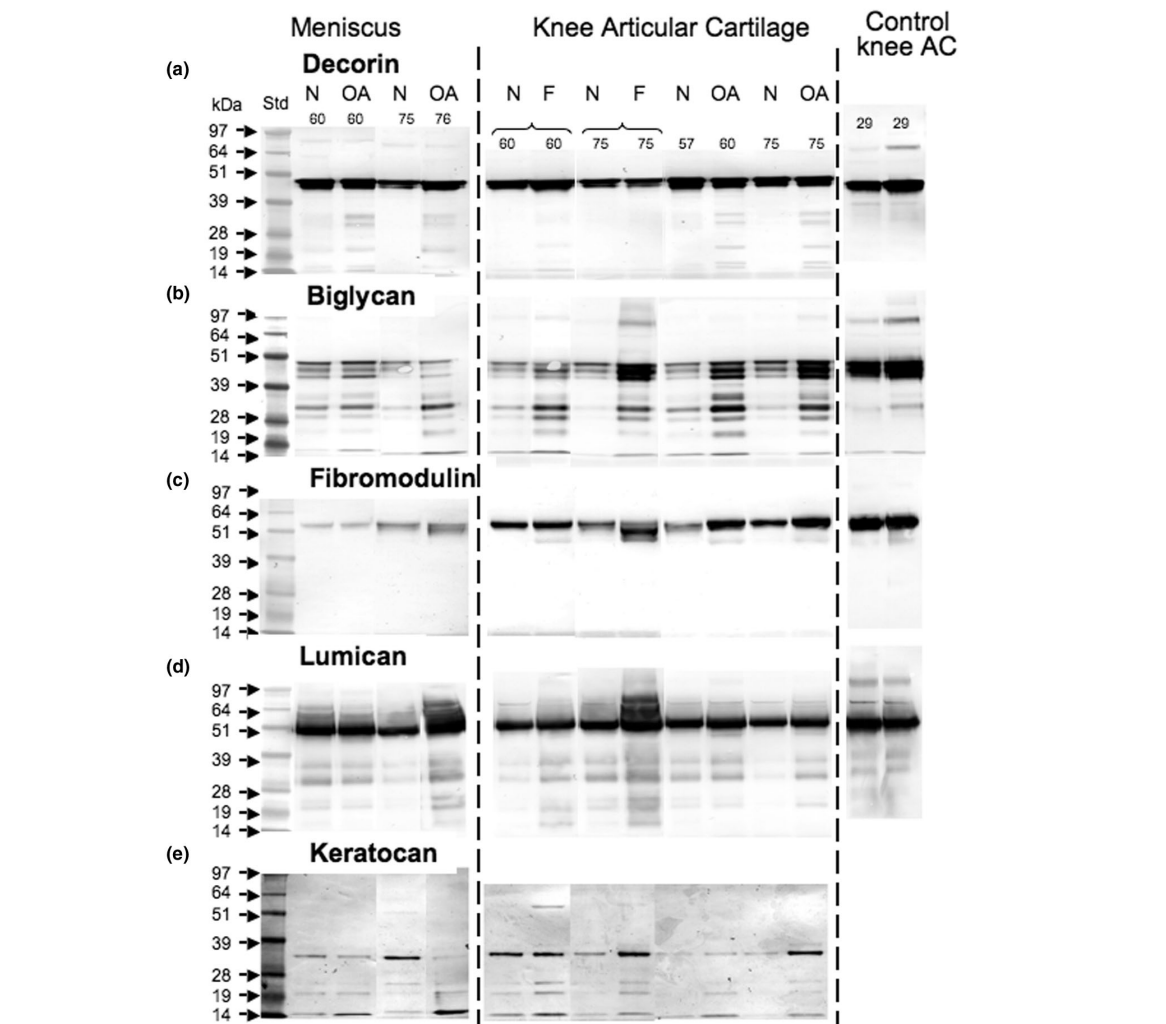
Identification of intact SLRP core proteins and fragments in knee articular cartilage. Presented is identification of intact small leucine-rich proteoglycan (SLRP) core proteins and fragments in age-matched macroscopically normal (N), osteoarthritic (OA) or fibrillated (F) knee articular cartilage (AC). We used affinity-purified anti-carboxyl-terminal SLRP antibodies (PR-84, PR-85, PR-184 and PR-353) and a monoclonal antibody to full-length keratocan core protein (KER-1) to perform Western blotting of samples separated by 4% to 12% Bis-Tris lithium dodecyl sulphate PAGE and blotted to nitrocellulose. All samples were pre-digested with chondroitinase ABC and keratanase-I before electrophoresis. The brackets above the lanes indicate that macroscopically normal and fibrillated articular cartilage were sampled from the same individual nonarthritic joint for this comparison. Extracts from an equivalent wet weight of tissue were loaded in each lane for comparison. The ages of tissue donors are indicated above each lane.

To enable comparison of multiple samples of both OA and normal tissues and to avoid any potential losses in SLRP core protein fragments (which are interactive with some > 100 kDa component in the extracts that apparently is resolved from the SLRP fragments during electrophoresis), we repeated these initial blotting experiments with individual tissue extracts that were not subjected to centrifugal diafiltration (Figure 2). We also examined extracts from age-matched macroscopically normal knee articular cartilage and from areas of the same joint displaying surface fibrillation, as well as extracts from normal young nondegenerate articular cartilage (Figure 2). These blots showed a similar range of SLRP fragments to those previously identified in the pooled tissue extracts (Figure 1b). In contrast to the pooled extract, however, similar levels of SLRP fragmentation were evident in meniscus and knee cartilage. In the meniscus there was a consistent increase in fragmentation of decorin but not the other SLRPs in OA versus age-matched normal joints. In contrast, in knee articular cartilage all SLRPs generally exhibited increased fragmentation in OA compared with similarly aged normal joints. Furthermore, in surface-fibrillated compared with intact cartilage from the same non-OA joints, there was a similar increase in fragmentation of all SLRPs (Figure 2). SLRP fragmentation levels in the fibrillated and OA knee articular cartilages were higher than the mature age-matched macroscopically normal tissue or normal young knee articular cartilage from two 29-year-old donors (Figure 2).

Six prominent decorin fragments (38,36, 25, 18, 16 and 14 kDa) were evident in the fibrillated and the degenerate cartilage specimens from the total knee replacement donors. Similar fragments were also identified in the meniscal samples from the total knee replacements, but these were largely undetectable in the 29-year-old normal cartilage samples (Figure 2). Fragmentation of biglycan in meniscus and cartilage exhibited a prominent triplet of 39 to 45 kDa and up to six variably distributed smaller molecular weight core protein species (16 to 35 kDa). Little fragmentation of fibromodulin was apparent, although one, almost full-length fibromodulin core protein fragment (approximately 49 kDa) was evident in fibrillated and OA cartilage but not meniscus. Lumican fragments in both meniscus and cartilage were of similar size, consisting of five catabolites ranging from 15 to 38 kDa. Similarly, prominent 35 to 37 kDa full-length keratocan core proteins and four core protein fragments (14 to 25 kDa) were evident, with a similar size distribution in meniscus and fibrillated and OA cartilage.

**Figure 3:**
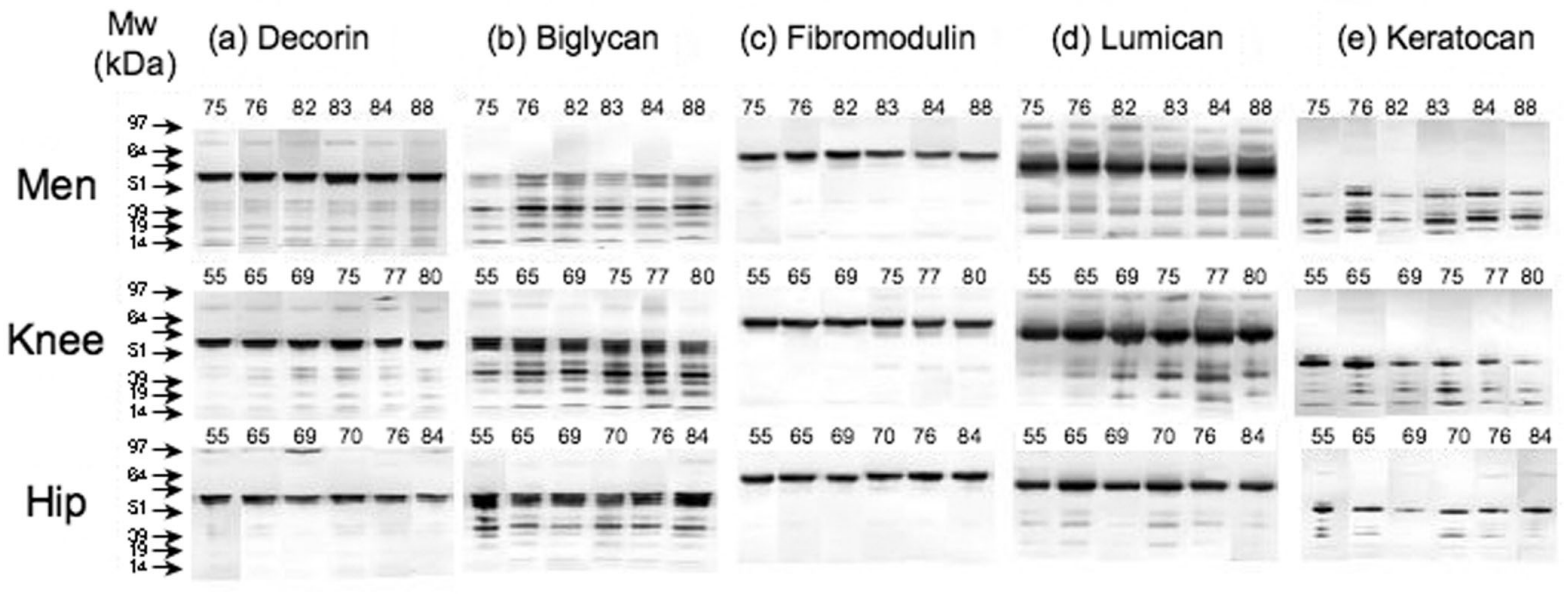
Identification of SLRP core protein fragmentation in meniscus, knee and hip articular cartilage. Presented is identification of small leucine-rich proteoglycan (SLRP) core protein fragmentation in 4 M GuHCl extracts of meniscus, knee and hip articular cartilage of individual tissue specimens from total knee or hip replacement patients. The ages (years) of each specimen are indicated at the top of each lane. Extracts from an equivalent wet weight of tissue were loaded in each lane. The samples were pre-digested with chondroitinase ABC and keratanase-I prior to electrophoresis. Migration positions of Novex SeeBlue2 Protein standards are indicated on the left hand side of each segment.

A third series of blots were undertaken on SLRP fragmentation using six representative individual tissue samples from the total knee replacement articular cartilage, hip replacement articular cartilage and knee joint menisci from the total knee replacement donors to determine whether there was an effect of age (Figure 3). The SLRP fragmentation patterns obtained were similar to those obtained earlier (Figures 1b and 2b). A noteable trend toward increased abundance of fragments of all SLRPs other than keratocan with age was evident in the knee articular cartilage samples but not the meniscus or hip cartilage. There were no fragments in any of the SLRPs that were specifically associated with ageing in the knee articular cartilage, but rather an increased staining of all fragments (Figure 3a–e). In the case of keratocan, the most notable change with age in the knee articular cartilage was a decrease in the intact core protein species (35 to 37 kDa; Figure 3e). As noted previously (Figure 1), there was generally less staining of all SLRP fragments in age-matched extracts of hip compared with knee OA articular cartilage.

## Discussion

This study has shown that SLRPs undergo extensive proteolysis in several diseased human and some normal age-matched musculoskeletal tissues. We have extended previous studies examining SLRP proteolysis that were restricted to arthritic knee cartilage [54-56] by showing the presence and catabolism of keratocan in this tissue. We have also compared and shown differences in the degree of SLRP catabolism in OA hip compared with knee cartilage. In addition we have, for the first time, described SLRP catabolism in the meniscus from OA and normal knees and in surface-fibrillated cartilage compared with intact tissue from nonarthritic joints.

One of the limitations of the study is that, in order to achieve sufficient tissue for analysis from individual OA joints, we generally pooled all available cartilage or meniscus for extraction. Thus, it was not possible to correlate the degree of gross or histological pathology with subsequent SLRP catabolism. Post-extraction proteolysis of SLRPs could account for some of the SLRP fragmentation observed in the present study, but this seems unlikely as proteinase inhibitors and ultra-pure de-ionized water were used in all steps. There was a disproportionate loss of SLRP catabolites in cartilage compared with meniscal extracts that were subjected to diafiltration, which may suggest interaction of SLRP fragments and removal with the aggrecan present in much higher levels in cartilage. Finally, we were only able to compare the molecular mass of the SLRP catabolites, because to date the actual cleavage sites in the core proteins and relevant neoepitope antibodies are not available.

Interestingly, we found that not all SLRPs exhibited a similar degree of fragmentation within the one tissue, and furthermore there were distinct differences between tissues and even between the same tissue from different joints (articular cartilage in knee versus hip). It is interesting to speculate that the differences observed between hip and knee cartilage could be associated with the generally more extensive pathology in hip joints, such that the residual cartilage may be in a more advanced stage of degeneration. It is also possible that some cartilage repair occurs in late stage OA and is more prevalent in the hip.

We did observe differences in SLRP proteolysis in the meniscus compared with the articular cartilage in OA joints, although these were more subtle than expected, given the disparity in cell type, matrix organization, matrix constituents (for example, collagen types) and vascularity of the two tissues. In all tissues examined in the present study the same molecular mass fragments were found for all SLRPs, suggesting that similar proteolytic events were responsible/occurring. When SLRP catabolites were present, this was also true of normal compared with arthritic joint tissues, again suggesting that the elevated breakdown of SLRPs in disease is due to the upregulation of the same enzymes which are responsible for the homeostatic turnover of these components in normal tissues. The exception was fibromodulin, in which a 49 kDa fragment was more evident in articular cartilage compared with meniscus. This suggests the presence of a specific proteolytic pathway or organization of fibromodulin in articular cartilage. Furthermore, this catabolite may be a useful distinguishing marker of degeneration in the two tissues. We were able to demonstrate an age-related pattern of SLRP proteolysis in OA knee articular cartilage but not meniscus (or hip). In the case of meniscus this may be because a more limited and elderly age range was available for comparison. It is unclear whether the increased SLRP proteolysis in OA knee articular cartilage is a true ageing phenomenon or whether joint disease was also worse in the older patients. We were only able to access a limited number of age-matched nonarthritic joints with a narrow age bracket (60 to 75 years) for comparison with OA, and we did not observe an age-associated pattern of SLRP fragmentation in these samples.

There was also a difference between knee meniscus and articular cartilage when SLRP proteolysis in normal and OA samples were compared. There was a consistent pattern of increased fragmentation of decorin, biglycan, fibromodulin, keratocan and, to a lesser extent, lumican in articular cartilage from age-matched OA compared with nonarthritic joints. This pattern only held true for decorin but not the other SLRPs in normal versus OA meniscus. This may be associated with the greater degree of degeneration in the articular cartilage compared with meniscus in OA joints. Alternatively, there may be an elevated basal (normal) level of SLRP cleavage in the aged meniscus, which would be consistent with the age-related increase in expression of decorin in meniscus but not articular cartilage previously reported [17]. It is difficult to explain why only decorin exhibited consistent increased fragmentation in meniscus in OA, but it may indicate differences between the SLRPs in proteolytic pathways, regulation of synthesis, or presentation/availability to enzymatic attack in the meniscus compared with articular cartilage.

The apparent limited catabolism of fibromodulin in all tissues may suggest that fibromodulin is more resistant to proteolysis than the other SLRPs or that fibromodulin degradation products retaining the carboxyl-terminus may not be stably retained in the tissue and are lost into the synovial fluid. Alternatively, it could be an artefact of using an antibody to the extreme carboxyl-terminus of the core protein. Once the LRLASLIEI carboxyl-terminal peptide sequence identified by Ab PR-184 is removed from the native fibromodulin core protein, it and any subsequent catabolites are no longer detectable with this antibody. Thus, it is possible that fibromodulin may be more extensively processed at the carboxyl-terminus in degenerate OA connective tissues compared with other SLRPs, in which extensive fragments were detected with antibodies to the carboxyl-terminus.

Decorin and fibromodulin were the SLRPs with the most distinctly increased fragmentation in OA and fibrillated articular cartilage from nondiseased joints compared with macroscopically normal articular cartilage from the age-matched donors. This suggests that proteolysis of these two SLRPs may be particularly associated with pathology, and that age-related articular cartilage fibrillation in apparently normal joints may involve similar proteolytic events as *bona fide *OA. Similar decorin fragments were also evident in the meniscal extracts from total knee replacement tissue donors but not the extracts of the menisci from the age-matched normal tissue donors. This coincident increase in decorin proteolysis in different tissues in joint pathology suggests that humoral factors such as interleukin-1 or tumour necrosis factor associated with disease may stimulate degradation of this SLRP and may imply that different cytokine levels in the knee and hip account for the site variations. Some of these decorin core protein species may therefore represent useful diagnostic biomarkers of joint disease. Fibromodulin catabolism on the other hand was more uniquely associated with articular cartilage and could be useful for tissue discrimination. In the case of biglycan and lumican in particular, despite an increase in fibrillated and OA articular cartilage, detectable levels of some of the fragments in macroscopically normal articular cartilage and meniscus indicate that these fragments are also associated with the normal turnover of these tissues. This would probably limit their utility as potential disease biomarkers.

A number of studies have examined the possible use of disease-associated protein fragments as biomarkers to evaluate articular cartilage metabolism or disease progression in spondyloarthritis and OA in humans and in animal models of OA [58-67]. In the present study we only identified fragments retained in the diseased tissues. However, with future determination of the specific cleavage site in the core protein that generate these catabolites, it will be possible to generate antibodies that recognize both the specific amino- and carboxyl-termini resulting from such proteolysis. These antibodies may permit discovery of potential biomarker peptides that are released into body fluids.

Melching and coworkers [40] demonstrated that recombinant aggrecanase-1 and aggrecanase-2 generated a major 27 kDa carboxy-terminal biglycan fragment *in vitro*, with cleavage being within the fifth leucine-rich domain. We also identified an approximately 27 kDa biglycan fragment in extracts of degenerate meniscus, knee and hip articular articular cartilage, consistent with ADAMTS (a disintegrin and metalloprotease domain with thrombospondin type I motifs) cleavage. Decorin, biglycan and fibromodulin can all also be degraded by MMP-13 *in vitro *with fragments that recognized by the same antibodies as used in the present study [41]. The 28 to 30 kDa catabolites of decorin and biglycan we observed are consistent with those generated by MMP-13. MMP-13 *in vitro *has also been shown to degrade fibromodulin attached to collagen with the generation of a 37 to 39 kDa carboxyl-terminal fragment, but fibromodulin in free solution was not degraded [38]. We failed to detect any significant 37 to 39 kDa fibromodulin catabolites expected from MMP-13 cleavage of this protein using PR-184. Importantly, the majority of the naturally occurring SLRP catabolites identified in human tissues in the present study do not correlate in size with fragments generated by *in vitro *digests with specific proteinases. This may indicate that enzymes other than those thus far studied *in vitro *are responsible for SLRP catabolism *in vivo *or that the cleavage sites and susceptibility may be different *in situ *as opposed to solution-phase digests. It is important that in the future the actual cleavages that occur in tissues are defined, in order to enable the enzymes responsible to be identified and potentially evaluated as targets for disease modification.

## Conclusion

In general, an extensive array of SLRP core protein fragments are present in degenerate knee articular cartilage and meniscus, but they were less prominent in degenerate hip articular cartilage. Specific decorin and fibromodulin core protein fragments, but not other SLRPs, were associated with the degenerate human meniscus and articular cartilage compared with nondiseased tissue.

Fibromodulin core protein fragmentation was far less evident than fragmentation of other members of the SLRP family. This may be because of fibromodulin being relatively resistant to proteolysis or, unlike other SLRPS studied, because the extreme carboxyl-terminus of fibromodulin containing the antibody recognition site is rapidly and/or extensively processed. The majority of the naturally occurring SLRP catabolites identified in human joint tissues in the present study do not correlate in size with fragments generated by *in vitro *digests with specific proteinases. This may indicate that enzymes other than those thus far studied *in vitro *are responsible for SLRP catabolism *in vivo *or that the cleavage sites and susceptibility may be different *in situ *as opposed to solution-phase digests. Future work may demonstrate some of the aforementioned SLRP core protein fragments as valuable biomarkers of joint disease progression. Identification of the enzymes responsible for their generation may also uncover useful targets for therapeutic intervention strategies for arthritic disorders.

## Abbreviations

MMP = matrix metalloproteinase; OA = osteoarthritis; PAGE = polyacrylamide gel electrophoresis; SD = standard deviation; SLRP = small leucine-rich proteoglycan; TBS = Tris-HCl 0.15 M NaCl (pH 7.2).

## Competing interests

The authors declare that they have no competing interests.

## Authors' contributions

JM was responsible for the day to day running of the study, experimental design and writing of the manuscript in conjunction with PJR and CBL. ESF undertook the collection of tissues, Western blotting and other incidental duties required for the day to day running of the project. PJR, CEH, BK and BC were involved in the supply of antibodies, review of drafts of the manuscript and technical support for antibody use in Western blotting applications. MMS was involved in tissue collection, manuscript revision. CBL provided intellectual overview and clinical relevance to the study.
